# An evidence review and nutritional conceptual framework for pre-eclampsia prevention

**DOI:** 10.1017/S0007114522003889

**Published:** 2023-09-28

**Authors:** Mai-Lei Woo Kinshella, Kelly Pickerill, Jeffrey N. Bone, Sarina Prasad, Olivia Campbell, Marianne Vidler, Rachel Craik, Marie-Laure Volvert, Hiten D. Mistry, Eleni Tsigas, Laura A. Magee, Peter von Dadelszen, Sophie E. Moore, Rajavel Elango, the PRECISE Conceptual Framework Working Group

**Affiliations:** 1 Department of Obstetrics and Gynaecology, BC Children’s Hospital Research Institute, University of British Columbia, Vancouver, BC V5Z 4H4, Canada; 2 Department of Women and Children’s Health, School of Life Course Sciences, Kings College London, London, UK; 3 Preeclampsia Foundation, Melbourne, FL, USA; 4 MRC Unit, The Gambia at the London School of Hygiene and Tropical Medicine, Fajara, The Gambia; 5 Department of Pediatrics, BC Children’s and Women’s Hospital, University of British Columbia, Vancouver, BC, Canada; 6 School of Population and Public Health, University of British Columbia, Vancouver, BC, Canada

**Keywords:** Pregnancy, Micronutrients, Maternal dietary patterns, Pre-eclampsia prevention, Conceptual framework, Evidence map

## Abstract

Pre-eclampsia is a serious complication of pregnancy, and maternal nutritional factors may play protective roles or exacerbate risk. The tendency to focus on single nutrients as a risk factor obscures the complexity of possible interactions, which may be important given the complex nature of pre-eclampsia. An evidence review was conducted to compile definite, probable, possible and indirect nutritional determinants of pre-eclampsia to map a nutritional conceptual framework for pre-eclampsia prevention. Determinants of pre-eclampsia were first compiled through an initial consultation with experts. Second, an expanded literature review was conducted to confirm associations, elicit additional indicators and evaluate evidence. The strength of association was evaluated as definite relative risk (RR) < 0·40 or ≥3·00, probable RR 0·40–0·69 or 1·50–2·99, possible RR 0·70–0·89 or 1·10–1·49 or not discernible RR 0·90–1·09. The quality of evidence was evaluated using Grading of Recommendations, Assessment, Development and Evaluation. Twenty-five nutritional factors were reported in two umbrella reviews and twenty-two meta-analyses. Of these, fourteen were significantly associated with pre-eclampsia incidence. Higher serum Fe emerged as a definite nutritional risk factors for pre-eclampsia incidence across populations, while low serum Zn was a risk factor in Asia and Africa. Maternal vitamin D deficiency was a probable risk factor and Ca and/or vitamin D supplementation were probable protective nutritional factors. Healthy maternal dietary patterns were possibly associated with lower risk of developing pre-eclampsia. Potential indirect pathways of maternal nutritional factors and pre-eclampsia may exist through obesity, maternal anaemia and gestational diabetes mellitus. Research gaps remain on the influence of household capacities and socio-cultural, economic and political contexts, as well as interactions with medical conditions.

Pre-eclampsia and other hypertensive disorders of pregnancy occur in 5–10 % of pregnancies and are associated with almost 30 000 maternal deaths, 416 000 stillbirths and 1·5–2 million neonatal deaths annually worldwide^(1–5)^. In addition, pre-eclampsia is associated with long-term adverse outcomes including high risk of future CVD, diabetes, dyslipidaemia and chronic kidney disease for the mother and higher risk for attention deficit/hyperactivity disorder, increased BMI and CVD among children exposed to pre-eclampsia^(6–10)^. Clinical and social risk factors for pre-eclampsia include prior pre-eclampsia, chronic hypertension, chronic kidney disease, obesity, primiparity, multifetal pregnancy, antiphospholipid antibody syndrome, conception by means of assisted reproductive technology, low socio-economic status and minority ethnic background^(11–13)^. The development of pre-eclampsia involves inadequate placentation, maternal inflammatory response, generalised endothelial dysfunction and high blood pressure^(13,14)^. Because nutrition is important for placentation and certain micronutrients have clinical antioxidant, anti-inflammatory and blood pressure regulating properties, maternal nutritional factors may play protective roles or heighten risk of developing pre-eclampsia^(15–18)^.

Nutritional factors are acknowledged as a key component in the 2016 WHO recommendations on routine antenatal care (ANC) for promoting maternal and child health and fourteen of the forty-nine recommendations relate to nutrition in pregnancy^(19)^. For pre-eclampsia prevention specifically in the WHO ANC and pre-eclampsia guidelines, nutritional interventions are limited to high dose (1·5–2 g daily) Ca supplementation in populations with low Ca dietary intake^(19,20)^. A review of clinical practice guidelines for pregnancy hypertension found that only aspirin and Ca were commonly recommended for the prevention of pre-eclampsia^(21)^. The tendency to focus on single nutrients obscures the complexity of possible interactions and causal pathways^(22)^, which may be important to understand given the complex nature of pre-eclampsia. The multi-factored evolution of risk with maternal nutrition and other clinical, biologic, social and environmental factors is not well understood, and this impacts capacities for developing prevention strategies.

This evidence review aims to compile definite, probable, possible and indirect nutritional determinants of pre-eclampsia reported in current literature, by magnitude of effect and quality of evidence in order to map a nutritional conceptual framework for pre-eclampsia prevention.

## Methods

We followed the methods of Hiatt *et al*.^(22)^ to develop a model of determinants using a systematic process. First, a broad group of pre-eclampsia experts were selected from the Epidemiology Working Group of the PREgnancy Care Integrating translational Science, Everywhere (PRECISE) Network to develop components for a working model of pre-eclampsia determinants divided into medical history, biomarkers, nutrition and social determinants quadrants^(23)^. Each of the quadrants was independently investigated and refined through a literature review to confirm associations, expand indicators and evaluate evidence. The present study focuses on the diet and nutrition quadrant.

### Search strategy

The diet and nutrition literature review involved a systematic search on the Cochrane Library and Medline Ovid from database inception to 11 October 2022, on Google Scholar and reference lists. Searches were conducted using the following terms: (pre-eclampsia OR preeclampsia) AND (pregnant OR pregnancy) AND (deficiency OR deficient OR nutrient OR nutrition OR supplement OR status).

The highest level of evidence supporting associations between risk factors and pre-eclampsia was identified in a hierarchical manner based on Grading of Recommendations, Assessment, Development and Evaluation (GRADE) standards^(24)^. We first sought umbrella reviews (systematic reviews of systematic reviews) reporting on nutritional factors and pre-eclampsia. If no relevant umbrella reviews were identified, then the process was expanded to identify relevant meta-analyses. High-quality meta-analyses, such as Cochrane systematic reviews, were prioritised where available. The process was repeated with individual randomised controlled trials (RCT), then large observational studies. We included observational studies with at least 1000 participants to attempt to be more representative of the general population and higher likelihood of sufficient statistical power to assess specific determinants^(11,25)^. We excluded smaller observational studies, case reports or series, qualitative reviews and editorials. Articles not written in English were excluded due to limited capacity of the review team to comprehensively search non-English databases.

### Study selection

Titles and abstracts of articles were screened to assess their eligibility based on study design (umbrella review, meta-analysis, RCT or large observational study), population (pregnant or women of reproductive age), exposure (nutritional biomarker or dietary pattern) or intervention (nutritional supplement or dietary intervention) and outcome (pre-eclampsia or known risk factor for pre-eclampsia). Potentially eligible studies reporting quantitative direct or indirect associations between nutritional factors and pre-eclampsia underwent full-text review. Articles were initially screened by MWK and then discussed with the British Columbia PRECISE Conceptual Framework Working Group (KP, SP, OC) for final decision on inclusion.

### Data extraction

Author, year, publication type (umbrella review, systematic review/meta-analysis, RCT, observational), risk factor, outcome, study design, number of participants, relative effect (95 % CI), variation between studies (*I* ^2^), strength of association and quality of evidence were extracted from each study onto a standardised, piloted data extraction form on Word (Microsoft Corporation). Relative effects of nutritional factors were extracted as relative risks (RR), OR, standardised mean difference (SMD) or calculated from the prevalence of pre-eclampsia (or known risk factor of pre-eclampsia for indirect associations) among women with and without the risk factor. Study characteristics necessary to assess evidence quality were also extracted. Data were extracted by MWK and quality checked by members of the British Columbia PRECISE Conceptual Framework Working Group (KP, SP, OC).

### Strength of association and quality of evidence assessment

Larger magnitude of effects is indicative of stronger evidence that the risk factor has an impact on the outcome and strength of association was assessed as definite (RR < 0·40 or ≥3·00), probable (RR 0·40–0·69 or 1·50–2·99), possible (RR 0·70–0·89 or 1·10–1·49) or not discernible/not significant (RR 0·90–1·09)^(22,26,27)^. Because pre-eclampsia occurs in less than 10 % of the exposed and unexposed populations, OR are a reasonable approximation of the RR and used interchangeably for the model^(28)^.

Quality of evidence was evaluated using GRADE and classified as high, moderate, low or very low^(24)^. Umbrella reviews, systematic reviews and RCT started as high certainty of evidence, while observational studies started as low certainty of evidence^(24)^. Studies were downgraded for potential risk for bias, inconsistency, indirectness, imprecision and publication bias and upgraded for large effect sizes and evidence of a dose–response^(24)^. Potential publication bias was indicated with an asymmetrical funnel plot^(24,29)^. Studies could be down or upgraded by one or two levels depending of the severity within each domain^(24)^.

Extracted data and GRADE evaluations were reviewed within the University of British Columbia PRECISE Conceptual Framework Working Group (MWK, KP, SP, OC) with oversight from nutrition experts (RE, SEM, HDM) and clinical experts (LAM, PvD) to ensure validity. Discrepancies were discussed until consensus was achieved. The model was refined based on input from the PRECISE Conceptual Framework Working Group. Nutritional factors were cross-checked with patient interests raised in The Preeclampsia Registry^(30)^. Priority areas raised by pre-eclampsia patients and families included folic acid, Fe, Na, vitamin D, Ca, fish oil and Mg.

## Results

**Fig. 1.**
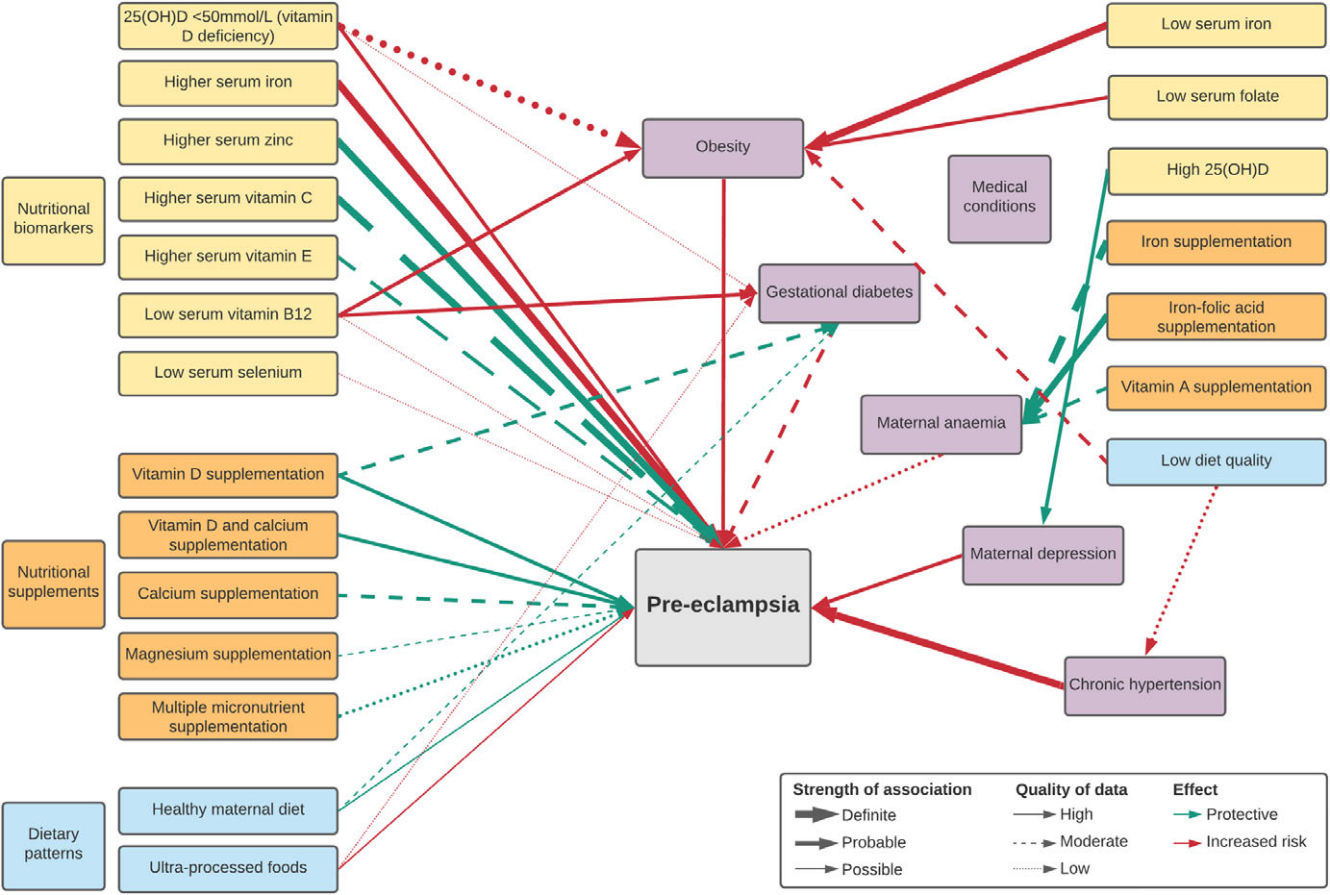
Map of significant direct and indirect nutritional risk factors for pre-eclampsia.

Overall, twenty-five nutritional factors were reported in two umbrella reviews^(12,31)^ and twenty-two meta-analyses^(32–54)^. These included eight biomarker levels (25(OH)D, Fe, Zn, Cu, Se, vitamins C, E and B_12_), fourteen nutritional supplementations (Ca and/or vitamin D, vitamin C and/or E, vitamin B_6_, Fe and/or folic acid, Mg, Zn, multiple micronutrients, *n*-3 fatty acids, balanced protein and energy), one dietary intervention (antenatal dietary counselling) and two dietary patterns (healthy maternal dietary pattern, ultra-processed foods). Fourteen factors were significantly associated with pre-eclampsia incidence (Table 1) while evidence did not support a significant association for eleven factors (online Supplementary Table S1). Additionally, there were fifteen nutritional factors potentially indirectly associated with pre-eclampsia incidence (Table 2) based on an umbrella review^(55)^, fifteen meta-analyses^(37,39,44,47,54,56–65)^ and three large cohort studies^(66–68)^. A summary of associations is illustrated in Fig. 1.

**Table 1.** Nutritional factors with significant associations with risk of developing pre-eclampsia (95 % confidence intervals)

Dietary or nutritional factor	Effect estimate	95 % CI	Number of studies	Number of participants	*I* ^2^	Quality of evidence
25(OH)D < 50 mmol/l^(12,35)^	OR 2·11	1·52, 2·94	6	2008	0 %	High
Serum Fe^(12,32)^	OR 9·97	4·00, 24·9	23	1912	96 %	High
Serum Zn* ^(12,33)^	OR 0·35	0·17, 0·68	14	1091	88 %	High
Serum vitamin C* ^(12,34)^	OR 0·37	0·22, 0·61	29	2777	91 %	Moderate
Serum vitamin E* ^(12,34)^	OR 0·46	0·27, 0·79	34	3398	93 %	Moderate
Serum vitamin B_12_^(36)^	WMD − 15·24 pg/ml	-27·52, −2·954	19	3211	98 %	Low
Serum Se^(51)^	SMD – 0·85	-1·46, −0·25	26	5583	96 %	Low
Vitamin D supplementation* ^(31,37)^	RR 0·62	0·43, 0·91	12	1353	0 %	High
Vitamin D and Ca supplementation* ^(31,37)^	RR 0·49	0·31, 0·77	3	1120	0 %	High
Ca supplementation^(52)^	RR 0·49	0·39, 0·61	30	20 445	59 %	Moderate
Mg supplementation* ^(53)^	RR 0·76	0·59, 0·98	7	2653	1 %	Moderate
Multiple micronutrient supplementation* ^(31)^	RR 0·40	0·27, 0·59	2	510	0 %	Low
Healthy maternal dietary pattern* ^(39)^	OR 0·78	0·70, 0·86	4	126 811	39 %	High
Ultra-processed foods dietary pattern^(54)^	OR 1·28	1·15, 1·42	4	112 307	0 %	High

RR, relative risk; WMD, weighted mean difference; SMD, standardised mean difference; NA, not applicable.

* Protective against developing pre-eclampsia.

**Table 2.** Nutritional factors with potential indirect associations with pre-eclampsia incidence via medical conditions (95 % confidence intervals)

Dietary or nutritional factor	Effect estimate	95 % CI	Number of studies	Number of participants	*I* ^2^	Quality of evidence
Maternal anaemia						
Vitamin A supplementation* ^(56)^	RR 0·64	0·43, 0·94	3	15 649	68 %	Moderate
Fe supplementation* ^(44)^	RR 0·30	0·19, 0·46	14	2199	80 %	Moderate
Fe–folic acid supplementation* ^(44)^	RR 0·34	0·21, 0·54	3	346	0 %	High
Gestational diabetes mellitus						
Vitamin D deficiency^(60)^	OR 1·43	1·23, 1·67	36	30 973	73 %	Low
Vitamin B_12_ < 200 pg/ml^(61)^	OR 1·81	1·25, 2·63	2	1129	0 %	High
Vitamin D supplementation* ^(37,55)^	RR 0·51	0·27, 0·97	4	446	0 %	Moderate
Healthy maternal dietary pattern* ^(39)^	OR 0·78	0·56, 0·99	5	6057	69 %	Moderate
Ultra-processed foods dietary pattern^(54)^	OR 1·48	1·17, 1·87	10	42 477	83 %	Low
Obesity						
Vitamin D deficiency^(63)^	OR 3·43	2·33, 5·06	15	13 209	81 %	Low
Serum folate <10 nmol/l^(66)^	aOR 2·03	1·35, 3·06	1	4243	N/A	High
Serum Fe <11 μmol/l^(66)^	aOR 3·26	2·09, 5·08	1	4243	N/A	High
Serum vitamin B_12_ < 203·3 pg/ml^(66)^	aOR 2·05	1·41, 2·99	1	4243	N/A	High
Low diet quality (Diet Quality Index for Pregnancy)^(67)^	aOR 1·76	1·24, 2·49	1	2394	N/A	Moderate
Maternal depression						
Maternal 25(OH)D* ^(65)^	OR 0·49	0·35, 0·63	6	10 317	82 %	High
Chronic hypertension						
Low diet quality (Healthy Eating Index)^(68)^	RR 2·67	1·67, 4·25	1	8259	N/A	Low

RR, relative risk.

* Protective effects.

### Definite associations

There were three nutritional factors with definite associations (Table 1). Higher serum Fe status was a risk factor while higher serum Zn was protective, based on high-quality evidence, and higher serum vitamin C was protective, based on moderate-quality evidence. High heterogeneity between studies was reported.

Women with pre-eclampsia had significantly higher serum Fe concentrations compared with healthy pregnant controls (SMD 1·27, 95 % CI (0·76, 1·78), 1912 participants, twenty-three studies, *I* ^2^ 96 %), largely assessed in the third trimester with high heterogeneity that remained after sensitivity analyses^(32)^. An umbrella review subsequently assessed that higher maternal serum Fe had almost ten times the odds of developing pre-eclampsia^(12)^. Increased maternal serum Fe levels among pre-eclamptic women were confirmed in another meta-analysis, which found higher levels among both Asian and European populations^(69)^.

Women with pre-eclampsia had lower Zn concentrations compared with healthy pregnant controls (SMD − 0·587, 95 % CI (−0·963, −0·212), 1091 participants, fourteen studies, *I* ^2^ 88 %), measured largely in the third trimester^(33)^. Significantly lower Zn concentrations among pre-eclamptic women compared with healthy controls were only found in Asia^(33,70,71)^ and sub-Saharan Africa^(72)^, not in other regions of the world.

Third-trimester concentrations of maternal serum vitamin C were significantly lower among women with pre-eclampsia (SMD − 0·56, 95 % CI (−0·83, −0·28), 2777 participants, twenty-nine studies, *I* ^2^ 91 %)^(34)^. Evidence for serum vitamin C had moderate certainty of evidence, due to the inclusion of some low-quality studies in meta-analyses and potential publication bias.

### Probable associations

There were six probable associations (Table 1). Protective effects of vitamin D on its own or co-supplemented with Ca were reported with high-quality evidence, maternal serum vitamin E status and Ca supplementation with moderate-quality evidence and maternal multiple micronutrient supplementation with low-quality evidence. Maternal vitamin D deficiency was a risk factor with high-quality evidence. High heterogeneity between studies was found for both Ca supplementation and serum vitamin E.

Maternal vitamin D deficiency, indicated by 25(OH)D < 50 mmol/l, was associated with increased odds of developing pre-eclampsia^(12,35)^. A larger effect of vitamin D deficiency compared with insufficiency (<75 mmol/l) suggests a potential dose–response, though confidence intervals overlap (deficiency OR 2·11, 95 % CI (1·52, 2·94) *v*. insufficiency OR 1·72, 95 % CI (1·11, 2·69))^(12,35)^. A subsequent meta-analysis with more included studies (twenty-one studies, 39 031 participants) also found a significant effect of vitamin D deficiency when measured around the second trimester among all populations except Oceanic groups^(73)^.

Third-trimester serum vitamin E was significantly lower among women with pre-eclampsia (SMD − 0·42, 95% CI (−0·72, −0·13), 3398 participants, thirty-four studies, *I* ^2^ 93 %)^(34)^. A recent large multicentre Chinese cohort study with 73 317 women found that low first-trimester serum vitamin E < 7·3 mg/l was also associated with higher risk of developing pre-eclampsia^(74)^. Moderate certainty of evidence resulted from the inclusion of some low-quality studies and potential publication bias.

Protective effects of vitamin D and Ca supplementation, each on their own, or supplemented together have been well documented in an umbrella review^(31)^, Cochrane systematic reviews^(37,38)^ and a network meta-analysis^(52)^. The majority of studies were conducted in low- and middle-income countries (LMIC); sensitivity analyses excluding high-income countries did not significantly change effects^(31)^. An earlier review of Ca supplementation in LMIC also found a significantly reduced risk of pre-eclampsia^(75)^. Higher dosages of vitamin D during pregnancy did not significantly increase benefit in comparison with lower dosages on the risk of developing pre-eclampsia (>600 mg/d *v*. ≤600 mg/d, ≥40 000 mg/d *v*. <40 000 mg/d), nor did commencement of supplementation either before or after 20 weeks gestation, though evidence was limited^(37,76)^. Both high (≥1 g/d) and low dose (<1 g/d) Ca supplementation had evidence of a strong beneficial effect, while Ca supplementation commencing early around the periconceptual period was not significant, based on very low-quality evidence. Ca supplementation overall had moderate certainty of evidence due to heterogeneity and potential publication bias.

A probable association between multiple micronutrient supplementation and pre-eclampsia prevention is based on low quality of evidence. Only two studies were found to report pre-eclampsia as an outcome, neither study using the United Nations standardised multiple micronutrients formulation^(31)^. While both included studies a significant beneficial effect, the selection criteria of study populations, timing and content of supplementation were different.

### Possible associations

There were five possible factors associated with pre-eclampsia prevention (Table 1). Lower maternal serum vitamin B_12_^(36)^ and Se^(51)^ may heighten risk for the development of pre-eclampsia, based on low certainty of evidence. Serum vitamin B_12_ was on average 15·24 pg/mL lower among women with pre-eclampsia when compared with those without^(36)^. Significantly lower Se concentrations among pre-eclamptic women compared with healthy controls were only found in African-based studies^(51)^. Mg supplementation may lower the odds of developing pre-eclampsia, based on moderate certainty of evidence. Pooled outcomes found a significant beneficial effect, though many of the individual Mg trials had non-significant results^(53)^.

Based on high-quality evidence, a healthy maternal dietary pattern characterised by high intake of fruits, vegetables, whole-grain foods, fish and poultry as highlighted in Mediterranean and New Nordic diets was associated with 22 % reduced odds of developing pre-eclampsia^(39)^. The review consisted of four, large, high-income country-based cohort studies: three from the Norwegian Mother and Child Cohort Study (MoBa) that assessed maternal diet in the second trimester^(77–79)^ and the Generation R Cohort Study from the Netherland with assessment at a median of 13·5 weeks^(80)^. A subsequent meta-analysis of LMIC-based studies found that adequate (≥1–3 servings/week) vegetable consumption reduced the odds of developing pre-eclampsia by 62 % (OR 0·38, 95 % CI (0·18, 0·80), four studies, 1391 participants, *I* ^2^ 85 %) and by 58 % with adequate (≥1–3 servings/week) fruit consumption (OR 0·42, 95 % CI (0·24, 0·71), five studies, 1676 participants, *I* ^2^ 79 %) compared with women with low or no consumption^(81)^. Conversely, maternal diets characterised by ultra-processed foods were associated with higher odds of developing pre-eclampsia, based on high-quality evidence and no heterogeneity between study results^(54)^.

### Not discernible

Based on moderate-quality evidence, antenatal dietary counselling was not significantly associated with pre-eclampsia prevention^(31)^ (online Supplementary Table S1). According to our methodology, there was no evidence supporting a direct association between maternal serum Cu^(12,40)^ or supplementation with any antioxidants^(41)^, vitamin B_6_^(49)^, vitamin C and/or E^(31,42,43)^, Fe and/or folic acid^(31,44)^, Zn^(31,46)^, *n*-3 fatty acids^(31,47)^ or protein-energy addition^(48)^ and pre-eclampsia prevention, all based on low to very-low quality evidence. See online Supplementary Table S2 for the GRADE assessment of each nutritional factor.

### Indirect associations

Nutritional factors with potential indirect associations with pre-eclampsia incidence via medical conditions are reported in Table 2. These include maternal anaemia (Hb <11 g/dl)^(82)^, particularly in the first trimester^(83)^ and when severe (Hb <7 g/dl)^(84)^, gestational diabetes mellitus (GDM)^(84)^, maternal overweight (BMI 25·0–29·9)^(11,85)^ and obesity (BMI ≥ 30)^(11,12,85)^, antenatal depression^(86)^ and chronic hypertension (pre-existing or hypertension diagnosed before 20 weeks)^(11,85)^ (see also online Supplementary Table S3).

Maternal anaemia may be lowered by Fe–folic acid supplementation and Fe supplementation based on high and moderate quality of evidence^(44)^, and possibly by vitamin A supplementation based on moderate quality of evidence^(56)^. There was no evidence for an effect of folic acid^(57)^, multiple micronutrient (any formulation, compared with Fe with or without folic acid)^(58)^, Ca^(59)^ or *n*-3 fatty acids^(47)^ supplementation (see online Supplementary Table S1).

Four nutritional factors were associated with risk of GDM. Based on high-quality evidence, low serum vitamin B_12_ increased risk of GDM^(61)^. Evidence around vitamin D is strengthened by corresponding findings that low maternal 25(OH)D increased risk of GDM^(60)^ while vitamin D supplementation was protective^(37,55)^. A healthy maternal dietary pattern may reduce GDM rates^(39)^, while conversely dietary patterns rich in ultra-processed foods may increase GDM rates^(54)^. Vitamin D and Ca co-supplementation^(37,55)^, *n*-3 supplementation^(47,55)^ and antenatal dietary counselling^(55,62)^ were not associated with rates of GDM.

Obesity was associated with five nutritional factors. Based on high-quality evidence, low serum Fe at 12–15 weeks gestation had a definite association with obesity, while low serum vitamin B_12_ and serum folate had moderate associations^(66)^. Low 25(OH)D (as defined by individual study authors for vitamin D deficiency) was a strong risk factor associated with over three-fold increased odds of obesity, but based on low-quality evidence due to high heterogeneity, potential publication bias and unclear quality of included studies^(63)^. Poor maternal diet quality (lowest tertile *v*. highest tertile on the Diet Quality Index for Pregnancy at 26–28 weeks gestation) had a probable association with obesity based on moderate-quality evidence^(67)^. Dietary diversity (among adult men and women)^(64)^ and maternal serum ferritin^(66)^ were not associated with obesity.

Based on high-quality evidence, women with the highest concentrations of maternal 25(OH)D significantly reduced the odds of antenatal and/or postnatal depression compared with women in the lowest category^(65)^. A large observational cohort study found that maternal low diet quality (lowest tertile *v*. highest quartile on the Healthy Eating Index) had a probable association with increased chronic hypertension^(67)^. No other nutritional factors for maternal depression and chronic hypertension were found according to our methodology.

## Discussion

### Summary of findings

Based on the magnitude of effect and evidence quality (online Supplementary Table S4), higher serum Fe was a strong nutritional risk factors for pre-eclampsia incidence across populations. Low serum Zn was a risk factor particularly in Asia and Africa, but Zn supplementation trials did not reduce pre-eclampsia incidence. Similarly, while there was some evidence of a protective effect of adequate maternal vitamin C and E, supplementation trials did not significantly reduce rates of pre-eclampsia.

Ca supplementation was by far the most studied nutrient in clinical trials to prevent pre-eclampsia, though high heterogeneity between study findings and potential publication bias led to an overall moderate certainty of evidence. Vitamin D supplementation with or without Ca tended to be investigated more recently. Though fewer trials than with Ca, vitamin D supplementation had higher certainty of evidence, with low heterogeneity and less potential publication bias. Certainty of the evidence is supported by complementary findings that vitamin D deficiency is associated with increased risk, while vitamin D supplementation reduced risk of developing pre-eclampsia, both supported by high-quality evidence. Vitamin D may also be indirectly associated with lower pre-eclampsia incidence through its protective effects on GDM, obesity and maternal depression.

Healthy maternal dietary patterns were possibly associated with lower risk of developing pre-eclampsia, with strong evidence from large, longitudinal studies, and evidence of larger effects in LMIC where malnutrition is prevalent. High-quality maternal diets were also protective against GDM, while low-quality diets increased risk of obesity and chronic hypertension. Evidence of healthy maternal dietary patterns is reinforced by increased risk of diet characterised by ultra-processed foods.

There was weak evidence for vitamin B_12_ deficiency as a risk factor, potentially through increased risk of GDM and obesity. Evidence was also limited for maternal Se levels and multiple micronutrient supplementation. Our evidence review did not find a significant association between overall antenatal dietary counselling and reduced risk of pre-eclampsia.

### Comparisons with existing literature and implications for practice

Previous umbrella reviews on pre-eclampsia determinants were not focused on maternal nutritional factors and/or only considered risk factors reported in systematic reviews with direct associations with pre-eclampsia^(12,31,85)^. The current review includes potential indirect pathways through medical conditions, particularly obesity, maternal anaemia and GDM. For example, maternal deficiencies in vitamin D, B_12_, folate and Fe were associated with obesity^(63,66)^, which was related to almost triple the risk of developing pre-eclampsia (RR 2·8, 95 % CI (2·6, 3·1))^(11)^. Whether maternal obesity increased the likelihood of nutritional deficiencies, nutritional deficiencies contributed to obesity, or both exacerbated each other remains unclear and methods to disentangle these complex relationships require high-quality data, often collected with longitudinal cohorts (over long periods of time) to assess^(87)^. Amplified by poor quality diets, obesity could be linked to reduced kidney function, altered metabolic processes and gut microbiota, thus preventing adequate nutrient absorption^(88,89)^. This fits with the Nutritional Conceptual Framework first outlined by the United Nation’s Children’s Fund (UNICEF) in 1990, which emphasised both the lack of adequate, nutritious food alongside frequent illness that impacted the capacity to absorb and utilise nutrients^(90,91)^. The discrepancies between low serum Zn, vitamin C and E as risk factors for developing pre-eclampsia, yet with the lack of evidence supporting their supplementation, suggest outstanding questions on absorption, utilisation, as well as the timing and dosage of supplementation.

Alongside Ca supplementation, which is recommended by WHO ANC and pre-eclampsia/eclampsia prevention and treatment guidelines^(19,20)^, Fe, vitamin D and overall healthy maternal diets were other maternal dietary factors that emerged strongly in our evidence review. High and low serum Fe were indicated as direct and indirect risk factors for pre-eclampsia, and recent reviews suggest non-linear relationship where both high and low Hb concentrations were associated with higher pre-eclampsia rates^(82,83)^. The WHO currently recommends Fe–folic acid supplementation for all pregnancies (antenatal multiple micronutrient supplements with Fe and folic acid in the context of research). However, understanding potential impacts on pre-eclampsia prevention is challenged by measurement gaps, which focus on perinatal outcomes^(92)^. For example, a Cochrane review on daily oral Fe supplementation in pregnancy found only four studies that reported pre-eclampsia in comparison with 11 reporting on low birth weight and 13 reporting on preterm birth^(44)^.While higher serum Fe was strongly associated with pre-eclampsia, findings largely result from hospital-based, case-controlled studies^(32)^. More research in women based studies is needed^(92)^ to investigate the conditions that lead to pathologically high Fe in women, whether genetic, environmental and/or potentially an effect exacerbated by pre-eclampsia.

A network meta-analysis found that vitamin D may be the best supplementation for lowering pre-eclampsia incidence^(93)^. In addition to supporting Ca absorption and regulation of blood pressure, vitamin D also has important roles in placental development and inflammation regulation^(94)^. Our review highlights potential indirect associations through GDM, obesity and maternal depression. Further quantification of the extent to which these mediated pathways explain the effect may be useful for further policy development and targeted interventions^(95)^. The WHO ANC guideline recently re-examined vitamin D supplementation, which noted 50 % reductions in the risk of pre-eclampsia and GDM. Supplementation was not recommended, in favour of instead promoting sunlight exposure and adequate nutrition^(96)^.

While our findings that healthy maternal dietary patterns contribute directly and indirectly to pre-eclampsia prevention support the WHO ANC guidelines on promoting healthy maternal diets, the feasibility for women to follow recommendations is a concern, particularly in LMIC^(31)^. It is noteworthy that a significant association between antenatal dietary counselling and pre-eclampsia prevention was not found in our review. In contrast, a beneficial effect was found in a previous meta-analysis of six high-income country-based studies of formal dietary counselling^(97)^, often facilitated by dietitians^(98–101)^. A review found that older, more educated women with higher incomes consistently scored higher on diet quality scores during pregnancy across different populations and settings^(102)^, which underscores the importance of socio-economic factors. Immediate causes of malnutrition are influenced by underlying household resources and socio-cultural, economic and political contexts in the UNICEF Nutritional Conceptual Framework^(90,91)^. With the focus on nutritional education during routine ANC, access to nutritious foods may be a barrier. Impact of nutritional interventions may be limited without a lifelong lens on improving nutrition of girls throughout their lives.

### Nutritional conceptual framework for pre-eclampsia and future directions in research

**Fig. 2.**
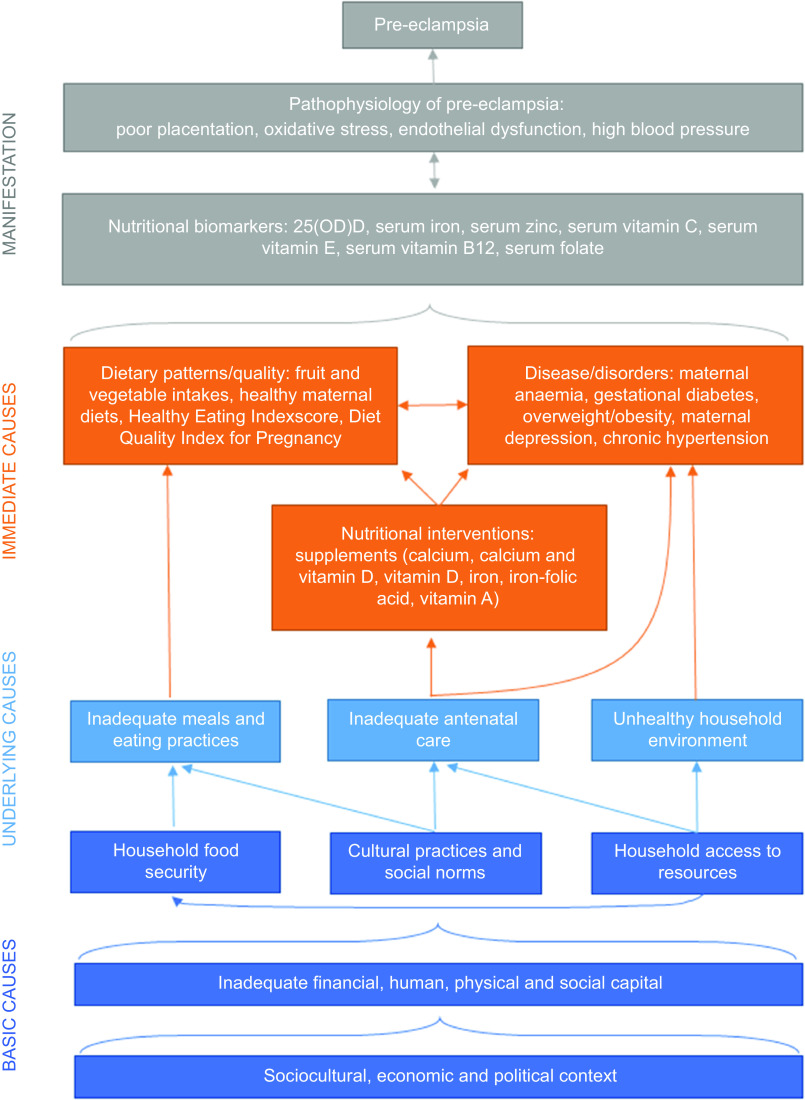
Nutritional conceptual framework for pre-eclampsia.

A working conceptual framework to understand dietary risk factors for pre-eclampsia adapted from the UNICEF Nutrition Conceptual Framework (Fig. 2) may help to showcase current gaps and guide future directions in research. There is a need to strengthen evidence on the relationships between nutritional factors, medical conditions and absorption, as well as associations between nutritional factors and underlying/basic causes. Framing nutritional factors by household capacities and socio-cultural, economic and political contexts may shed light on the underlying baseline risks that modify the efficacy of micronutrient supplementation trials, which has largely resulted in few significant effects with the exception of Ca and vitamin D^(31)^. With growing understanding that increasing nutritional levels may only be effective in pre-eclampsia prevention when baseline levels are low^(52,74)^, there is a need for future reviews to describe differences between high-income countries and LMIC more explicitly and future research in resource-limited settings to further tease out the impact of underlying risk factors.

### Strengths and limitations

Strengths of our analysis include consultation of nutrition and pre-eclampsia experts to guide the development and refinement of variables alongside a systematic methodology following Hiatt *et al*.^(22)^ and GRADE standards^(24)^ to compile and critically appraise evidence. Prioritisation of umbrella reviews and Cochrane systematic reviews supported a wide coverage of available studies globally, with rigorous evaluation of their potential risk of bias.

While our evidence review had several quality assurance mechanisms, some limitations included exclusion of non-English studies and lack of double extraction. Additionally, evaluating evidence was challenged by differing capacities to investigate variables. Nutritional biomarkers can be evaluated using objective blood tests, which lends to more certainty of the evidence but are limited by the small panel of biomarkers that researchers select to assess. Supplementation and antenatal counselling interventions are impacted by implementation quality and scope. Dietary patterns and social determinants are often limited by self-reported data, differing definitions between studies and many exposures are not feasible or ethical to evaluate using RCT. Our exclusion of observational studies with less than 1000 participants may have missed some variables. For example, investigating nutritional determinants of obesity among women of reproductive age was challenged by the lack of large cohort studies on the topic. Non-significant results may be inconclusive as there was a lack of high-quality evidence. Compiling evidence around nutritional factors may benefit from more standardised definitions for exposures, outcomes and statistical analyses particularly in observational studies.

### Conclusion

Vitamin D, Ca and Fe are strong nutritional factors, both directly and indirectly involved in pre-eclampsia prevention. Healthy maternal diet is a promising approach but more research is needed to understand how best to promote such diets, especially in resource-constrained settings. Zn, vitamins C, E and B are potential areas warranting further investigation, particularly in deficient populations, and around timing of intervention during placental development. A more comprehensive assessment of a full range of nutritional biomarkers is required in future research. We recommend a two-pronged approach: first, to investigate underlying social factors that influence food accessibility and dietary choice and second, to understand nutrient absorption and the impact of co-morbidities, including obesity, GDM and maternal anaemia, as potential mediating factors between maternal dietary intake and risk of developing pre-eclampsia. While WHO guidelines acknowledge the importance of maternal diets for the well-being of mothers and children, nutritional recommendations for pre-eclampsia prevention are currently limited. Recommendations can be strengthened with further evidence-based research into a number of promising areas.

## Supplementary material

For supplementary material accompanying this paper visit https://doi.org/10.1017/S0007114522003889. click here to view supplementary material
