# Supplementary material for: An evidence review and nutritional conceptual framework for pre-eclampsia prevention
Source: Br J Nutr. 2022 Dec 9;130(6):1065–76. doi: 10.1017/S0007114522003889 (PMC10442797; doi:10.1017/S0007114522003889)
Supplement: Supplementary file 1 [file S0007114522003889sup001.pdf]

Supplementary appendix

Tables and figures	Page number
Table S1: Table of results	Page 2
Table S2: GRADE quality of evidence assessment	Page 10
Table S3: Medical risk factors for pre-eclampsia incidence	Page 26
Table S4: Nutritional factors with direct associations with pre-eclampsia development, by strength of association and certainty of evidence	Page 27

Table S1: Table of results

Risk factor category	Author, Year, Type	Risk factor (arrow start)	Outcome (arrow end)	Study design	Studies	No. of participants	Relative effect (95% CI)	Variation between studies (I^2)	Strength of association	Quality of evidence (GRADE)
Biomarker	Wei 2013, SR / Giannakou 2017 UR	Maternal 25(OH)D < 50 mmol/L	PE	Case-control, cross-sectional, cohort	6	2008	2.11 (1.52–2.94) [1]	0%	Definite	High
Biomarker	Song 2015, SR / Giannakou 2017 UR	Maternal serum iron	PE	Case-control, cross-sectional	23	1912	OR 9.97 (4.00–24.9) [1]	96%	Definite	High
Biomarker	Ma 2015, SR / Giannakou 2017 UR	Maternal serum zinc	PE	Case-control, cross-sectional	14	1091	OR 0.35 (0.17–0.68)[1]	88%	Definite	High
Biomarker	Cohen 2015, SR / Giannakou 2017 UR	Maternal serum vitamin C	PE	Case-control, cross-sectional, cohort, RCT sub-study	29	2777	OR 0.37 (0.22–0.61) [1]	91%	Definite	Moderate
Biomarker	Cohen 2015, SR / Giannakou 2017 UR	Maternal serum vitamin E	PE	Case-control, cross-sectional, cohort, RCT sub-study	34	3398	OR 0.46 (0.27–0.79) [1]	93%	Probable	Moderate
Biomarker	Mardali 2020, SR	Maternal serum vitamin B12	PE	Case control	19	3211	WMD -15.24 pg/mL (-27.52 to -2.954) [1]	98%	N/A	Low
Biomarker	Hamdan 2022, SR	Maternal serum selenium	PE	Case control, cohort	26	5583	SMD - 0.85 (-1.46 to - 0.25) [1]	96%	N/A	Low

Biomarker	Fan 2016, SR / Giannakou 2017 UR	Maternal copper level	PE	Case-control, cross-sectional	12	905	OR 1.86 (0.41–8.51) [1]	97%	Not significant	Very low
Nutritional supplement	Kinshella 2021, UR	Vitamin D	PE	RCTs	12	1353	RR 0.62 (0.43–0.91) [1]	0%	Possible	High
Nutritional supplement	Palacios 2019, Cochrane SR	Vitamin D	PE	RCTs	4	499	RR 0.48 (0.30-0.79) [1]	0%	Possible	High
Nutritional supplement	Kinshella 2021, UR	Vitamin D and calcium	PE	RCTs	3	1120	RR 0.49 (0.31-0.77)[1]	0%	Possible	High
Nutritional supplement	Palacios 2019, Cochrane SR	Vitamin D and calcium	PE	RCTs	4	1174	RR 0.50 (0.32-0.78	0%	Possible	High
Nutritional supplement	Kinshella 2022, SR	Calcium	PE	RCTs	30	20445	RR 0.49 (0.39 – 0.61) [1]	59%	Probable	Moderate
Nutritional supplement	Hofmeyr 2018, Cochrane SR	High dose calcium (≥1g/d)	PE	RCTs	13	15730	RR 0.45 (0.31-0.65) [1]	76%	Possible	Low
Nutritional supplement	Hofmeyr 2018, Cochrane SR	Low dose calcium (<1g/d)	PE	RCTs	9	2234	RR 0.38 (0.28-0.52) [2]	0%	Definite	Moderate
Nutritional supplement	Hofmeyr 2019, Cochrane SR	Periconceptional calcium	PE	RCTs	1	579	RR 0.80 (0.61-1.06) [2]	NA	Not significant	Very low
Nutritional supplement	Rumbold 2008, Cochrane SR	Any antioxidant(s)	PE	RCTs	9	5446	RR 0.73 (0.51-1.06) [1]	56%	Not significant	Low
Nutritional supplement	Salam 2015, Cochrane SR	Vitamin B6 (oral)	PE	RCTs	2	1197	RR 1.71 (0.85-3.45) [2]	NA	Not significant	Low
Nutritional supplement	Kinshella 2021, UR	Vitamin C and/or E	PE	RCTs	19	24819	RR 0.96 (0.89–1.04) [1]	33%	Not significant	Low

Nutritional supplement	Rumbold 2015a, Cochrane SR	Vitamin C	PE	RCTs	16	21956	RR 0.92 (0.80-1.05) [1]	41%	Not significant	Very low
Nutritional supplement	Rumbold 2015b, Cochrane SR	Vitamin E	PE	RCTs	14	20878	RR 0.91 (0.79-1.06) [1]	48%	Not significant	Very low
Nutritional supplement	Kinshella 2021, UR	Iron and/or folic acid	PE	RCTs	6	4454	RR 0.99 (0.67–1.47) [1]	0%	Not significant	Low
Nutritional supplement	Peña-Rosas 2015, Cochrane SR	Any iron	PE	RCTs	4	1704	RR 1.63 (0.87-3.07) [1]	0%	Not significant	Low
Nutritional supplement	Peña-Rosas 2015, Cochrane SR	Iron-folic acid	PE	RCTs	1	48	RR 3.0 (0.13-70.16) [1]	NA	Not significant	Very low
Nutritional supplement	Yuan 2022, SR	Magnesium	PE	RCTs	7	2653	RR 0.76 (0.59-0.98) [1]	1%	Possible	Moderate
Nutritional supplement	Kinshella 2021, UR	Zinc	PE	RCTs	5	2518	RR 1.31 (0.82–2.10) [1]	39%	Not significant	Low
Nutritional supplement	Ota 2015b, Cochrane SR	Zinc	PE or PIH	RCTs	7	2975	RR 0.83 (0.64 - 1.08) [2]	26%	Not significant	Low
Nutritional supplement	Kinshella 2021, UR	Multiple micronutrient	PE	RCTs	2	510	RR 0.40 (0.27–0.59) [1]	0%	Probable	Low
Nutritional supplement	Kinshella 2021, UR	Omega-3 long chain poly-unsaturated fatty acids	PE	RCTs	18	7166	RR 0.87 (0.71–1.07) [1]	9%	Not significant	Moderate
Nutritional supplement	Middleton 2019, Cochrane SR	Omega-3 long chain poly-unsaturated fatty acids	PE	RCTs	20	8306	RR 0.84 (0.69-1.01) [2]	13%	Not significant	Low
Nutritional supplement	Ota 2015a, Cochrane SR	Balanced protein and energy	PE	RCTs	2	463	RR 1.48 (0.82-2.66) [2]	NA	Not significant	Low

Dietary intervention	Kinshella 2021, UR	Antenatal dietary counselling	PE	RCTs	15	8087	RR 0.97 (0.84–1.14) [1]	6%	Not significant	Moderate
Dietary pattern	Kibret 2018, SR	Healthy maternal dietary pattern (high intake of fruits, vegetables, whole-grain foods, fish and poultry)	PE	Cohort	4	126811	OR 0.78 (0.70-0.86) [1]	39%	Possible	High
Dietary pattern	Paula 2022, SR	Ultra-processed foods-rich diet (included at least one ultra processed food defined by the NOVA Food Classification System including fast foods, junk foods, processed meats, soft drinks, confectionaries, pizzas, hamburgers, candies and sweets, sweetened beverages and cookies, or a “Western diet” characterized by higher intake of energy-dense and processed foods)	PE	Cohort	4	112 307	OR 1.28 (1.15 – 1.42) [1]	0%	Possible	High
**INDIRECT ASSOCIATIONS**										
Nutritional supplement	McCauley 2015, Cochrane SR	Vitamin A	Maternal anaemia	RCTs	3	15649	RR 0.64 (0.43-0.94) [1]	68%	Probable	Moderate
Nutritional supplement	Peña-Rosas 2015, Cochrane SR	Iron	Maternal anaemia	RCTs	14	2199	RR 0.30 (0.19-0.46) [1]	80%	Definite	Moderate

Nutritional supplement	Peña-Rosas 2015, Cochrane SR	Iron-folic acid	Maternal anaemia	RCTs	3	346	RR 0.34 (0.21-0.54) [1]	0%	Definite	High
Nutritional supplement	Lassi 2013, Cochrane SR	Folic acid	Maternal anaemia	RCTs	8	4149	RR 0.62 (0.35-1.10) [1]	90%	Not significant	Very low
Nutritional supplement	Keats 2019, Cochrane SR	Multiple micronutrient (vs iron with or without folic acid)	Maternal anaemia	RCTs	9	60822	RR 1.04 (0.94-1.15) [1]	50%	Not significant	Low
Nutritional supplement	Buppasiri 2015, Cochrane SR	Calcium	Maternal anaemia	RCTs	1	1098	RR 1.04 (0.90-1.22) [2]	N/A	Not significant	Low
Nutritional supplement	Middleton 2019, Cochrane SR	Omega-3	Maternal anaemia	RCTs	1	846	RR 1.16 (0.91-1.48) [2]	N/A	Not significant	Low
Biomarker	Tripathi 2019, SR	Maternal vitamin D deficiency (not defined)	GDM	Case-control, cross-sectional, cohort	36	30973	OR 1.43 (1.23–1.67) [1]	73%	Possible	Low
Biomarker	Kouroglou 2019, SR	Maternal vitamin B12 <200 pg/mL	GDM	Case control, cohort	2	1129	OR 1.81 (1.25–2.63) [2]	0%	Probable	High
Nutritional supplement	Palacios 2019, Cochrane SR/ Griffith 2020, Cochrane UR	Vitamin D	GDM	RCTs	4	446	RR 0.51 (0.27-0.97) [1]	0%	Probable	Moderate

Nutritional supplement	Palacios 2019, Cochrane SR/ Griffith 2020, Cochrane UR	Vitamin D and calcium	GDM	RCTs	1	54	RR 0.33 (0.01-7.84) [1]	N/A	Not significant	Very low
Nutritional supplement	Middleton 2019, Cochrane SR/ Griffith 2020, Cochrane UR	Omega-3	GDM	RCTs	12	5235	RR 1.02 (0.83-1.26) [2]	0%	Not significant	Low
Dietary intervention	Tieu 2017, Cochrane SR / Griffith 2020, Cochrane UR	Antenatal dietary counselling	GDM	RCTs	5	1279	RR 0.60 (0.35-1.04) [1]	56%	Not significant	Very low
Dietary pattern	Kibret 2018, SR	Healthy maternal dietary pattern (high intake of fruits, vegetables, whole-grain foods, fish and poultry)	GDM	Cohort	5	6057	OR 0.78 (0.56-0.99) [1]	69%	Possible	Moderate
Dietary pattern	Paula 2022, SR	Ultra-processed foods-rich diet (included at least one ultra processed food defined by the NOVA Food Classification System including fast foods, junk foods, processed meats, soft drinks, confectionaries, pizzas, hamburgers, candies and sweets, sweetened beverages and cookies, or a "Western diet" characterized by	GDM	Cohort	10	42 477	OR 1.48 (1.17 – 1.87) [1]	83%	Possible	Low

		higher intake of energy-dense and processed foods)								
Biomarker	Yao 2015, SR	Maternal serum vitamin D 25(OH)D (as defined by individual study authors for deficiency)	Obesity	Case control and cross sectional	15	13209	OR 3.43 (2.33-5.06) [1]	81%	Definite	Low
Biomarker	Scholing 2018, cohort study	Pre-pregnancy obesity (BMI ≥30)	Maternal serum folate <10 nmol/l (12-15 weeks gestation)	Cohort study	1	4243	aOR 2.03 (1.35-3.06) [6]	NA	Probable	High
Biomarker	Scholing 2018, cohort study	Pre-pregnancy obesity (BMI ≥30)	Maternal serum ferritin <15 µg/l (12-15 weeks gestation)	Cohort study	1	4243	aOR 0.96 (0.62-1.51) [6]	NA	Not significant	Very low
Biomarker	Scholing 2018, cohort study	Pre-pregnancy obesity (BMI ≥30)	Maternal serum iron <11 µmol/l (12-15 weeks gestation)	Cohort study	1	4243	aOR 3.26 (2.09-5.08) [6]	NA	Definite	High
Biomarker	Scholing 2018, cohort study	Pre-pregnancy obesity (BMI ≥30)	Maternal serum vitamin B12 <203.3 pg/ml (12-15 weeks gestation)	Cohort study	1	4243	aOR 2.05 (1.41-2.99) [6]	NA	Probable	High
Dietary pattern	Salehi-Abargouei 2016, SR	Dietary diversity (highest vs lowest DDS)	Obesity	Cross sectional studies	8	6091	OR 0.72 (0.45–1.16) [1]	81%	Not significant	Very low

Dietary pattern	Laraia 2007, cohort study	Pre-pregnancy obesity (BMI >29, vs underweight BMI <19.8)	Lowest diet quality tertile (Diet Quality Index for Pregnancy at 26-28 weeks gestation)	Cohort study	1	2394	aOR 1.76 (1.24–2.49) [7]	NA	Probable	Moderate
Biomarker	Scholing 2018, cohort study	Pre-pregnancy overweight (BMI 25–29.9)	Maternal serum folate <10 nmol/l (12-15 weeks gestation)	Cohort study	1	4243	aOR 1.38 (1.03-1.85) [6]	NA	Possible	Moderate
Biomarker	Scholing 2018, cohort study	Pre-pregnancy overweight (BMI 25–29.9)	Maternal serum ferritin <15μg/l (12-15 weeks gestation)	Cohort study	1	4243	aOR 0.96 (0.73-1.27) [6]	NA	Not significant	Very low
Biomarker	Scholing 2018, cohort study	Pre-pregnancy overweight (BMI 25–29.9)	Maternal serum iron <11 nmol/l (12-15 weeks gestation)	Cohort study	1	4243	aOR 1.45 (1.02-2.07) [6]	NA	Possible	Moderate
Biomarker	Scholing 2018, cohort study	Pre-pregnancy overweight (BMI 2–29.9)	Maternal serum vitamin B12 <203.3 pg/ml (12-15 weeks gestation)	Cohort study	1	4243	aOR 1.38 (1.08-1.77) [6]	NA	Possible	Moderate
Dietary pattern	Laraia 2007, cohort study	Pre-pregnancy overweight (BMI>26–29, vs underweight BMI <19.8)	Lowest diet quality tertile (Diet Quality Index for Pregnancy at 26-28 weeks gestation)	Cohort study	1	2394	aOR 1.31 (0.87–1.99) [7]	NA	Not significant	Low

Biomarker	Tan 2019, SR	Maternal serum vitamin D 25(OH)D (highest vs lowest category in individual studies)	Maternal depression	Cohort, case–control and cross-sectional studies	6	10317	OR 0.49 (0.35-0.63) [1]	82%	Probable (*significant effect for antenatal and/or postnatal depression)	High
Dietary pattern	Yee 2020, cohort study	Healthy Eating Index (lowest vs highest quartile)	Chronic hypertension	Cohort study	1	8259	RR 2.67 (1.67-4.25)	NA	Probable	Low

PE – pre-eclampsia; PIH – pregnancy induced hypertension; GH – gestational hypertension SR – systematic review; UR – umbrella review; RCT – randomized controlled trial; OR – odds ratio; RR – relative risk; WMD – weighted mean difference; SMD – standardized mean difference; NA – not applicable

[1] random effects model
[2] fixed effects model

Table S2: GRADE quality of evidence assessment

**Reference**			**Certainty assessment**						**Effect**		**Certainty**
**Author, year**	**Association evaluated**	**# studies**	**Study design**	**Risk of bias**	**Inconsistency**	**Indirectness**	**Imprecision**	**Publication bias**	**Relative (95% CI)**	**Dose response**	
			High: RCT, umbrella review, systematic review<br><br>*Low: Observational study > 1000 participants*	*1 ↓ lack of inclusion or discussion of a sensitivity analysis AND/OR*<br>*1 ↓ Study limitations*	*1 ↓* $I^2 > 50\%$	*1 ↓ Serious Indirectness: Study excludes women from population. OR*<br>*2 ↓ Very serious indirectness*	*1 ↓Sample size <1000 (unless SR) or not reported AND/OR*<br>*1 ↓ CI crosses 1 (line of no effect)*	*1 ↓ asymmetrical funnel plots or no mention of publication bias AND/OR*<br>*1 ↓ evidence of very strong publication bias*	*1 ↑ Large effect: RR/OR > 2-5 or 0.5 – 0.2*<br>*2 ↑ Very large effect: > 5 or RR/OR < 0.2*	*1 ↑if existent*	*High, moderate or low*
Wei 2013	25 (OH) D <50 mmol/L on PE	6	SR	Not serious (studies with high risk of bias excluded as per protocol)	Not serious (1^2 28 %)	Not serious (pregnant women)	Not serious (sample >1,000, 95%CI does not cross 1)	Not serious (symmetrical funnel plot)	OR 2.11 (1.52–2.94) (+1)	Yes; <50 mmol/L larger effect than <75 mmol/L (+1)	High
Song 2015	Serum iron on PE	23	SR	Not serious (no studies rated low quality on NOS)	Serious (1^2 96%)	Not serious (pregnant women)	Not serious (sample >1,000, 95%CI does not cross 1)	Serious (Egger test significant)	OR 9.97 (4.00–24.9) (+2)	None reported	High
Ma 2015	Serum zinc on PE	14	SR	Not serious (all studies rated as 7 or 8 on NOS)	Serious (1^2 88%)	Not serious (pregnant women)	Not serious (sample >1,000, 95%CI does not cross 1)	Not serious (symmetrical funnel plot)	OR 0.35 (0.17–0.68) (+2)	None reported	High
Cohen 2015	Serum vitamin C on PE	29	SR	Serious risk of bias (some low quality studies according to NOS)	Serious (1^2 91%)	Not serious (pregnant women)	Not serious (sample >1,000, 95%CI does not cross 1)	Serious (asymmetrical funnel plot)	OR 0.37 (0.22–0.61) (+2)	None reported	Moderate

Cohen 2015	Serum vitamin E on PE	34	SR	Serious risk of bias (some low quality studies according to NOS)	Serious (1^2 93%)	Not serious (pregnant women)	Not serious (sample >1,000, 95%CI does not cross 1)	Serious (asymmetrical funnel plot)	OR 0.46 (0.27–0.79) (+2)	None reported	Moderate
Mardali 2020	Maternal serum vitamin B12 on PE	19	SR	Not serious (no studies rated low quality on NOS)	Serious (1^2 98%)	Not serious (pregnant women)	Not serious (sample >1,000, 95%CI does not cross 1)	Serious (asymmetrical funnel plot)	WMD - 15.24 pg/mL (- 27.52 to - 2.954)	None reported	Low
Hamdan 2022	Maternal serum selenium on PE	26	SR	Not serious (no studies rated low quality on NOS)	Serious (1^2 96%)	Not serious (pregnant women)	Not serious (sample >1,000, 95%CI does not cross 1)	Serious (asymmetrical funnel plot)	SMD - 0.85 (-1.46 to -0.25)	None reported	Low
Fan 2016	Maternal copper level on PE	12	SR	Serious (inclusion of sensitivity analysis but no evaluation of individual study limitations)	Serious (1^2 97%)	Not serious (pregnant women)	Serious (sample >1,000 but 95%CI crosses 1)	Not serious (Egger test not significant)	OR 1.86 (0.41–8.51)	None reported	Very low
Kinshella 2021	Vitamin D supplement on PE	12	UR	Not serious (majority studies low/unclear RoB)	Not serious (I^2 0%)	Not serious	Not serious (large sample, 95%CI does not cross 1)	Not serious (symmetrical funnel plot)	RR 0.62 (0.43-0.91)	Not reported	High
Palacios 2019	Vitamin D supplement on PE	4	SR	Serious risk of bias (high risk of selection bias and attrition bias, lack of blinding of participants and personnel in Sablok 2015)	Not serious (1^2 0%)	Not serious (pregnant women)	Not serious (sample <1,000 but SR, 95%CI does not cross 1)	NA (less than 10 studies included in meta-analysis)	RR 0.48 (0.30-0.79) (+1)	Not reported	High

Kinshella 2021	Calcium and vitamin D supplement on PE	3	RCTs	Serious (Less than half low/unclear RoB)	Not serious (I^2 0%)	Not serious	Not serious (large sample, 95%CI does not cross 1)	N/A	RR 0.49 (0.31-0.77)	Not reported	High
Palacios 2019	Calcium and vitamin D supplement on PE	4	SR	Serious risk of bias (lack of blinding in Asemi 2012, Marya 1987 and Taherian 2002; high risk of attrition bias and reporting bias in Marya 1987)	Not serious (1^2 0%)	Not serious (pregnant women)	Not serious (sample >1000, 95%CI does not cross 1)	NA (less than 10 studies included in meta-analysis)	RR 0.50 (0.32-0.78) (+1)	Not reported	High
Kinshella 2022	Calcium supplement on PE	30	SR	Not serious (Majority studies low/unclear RoB)	Serious (I^2 59%)	Not serious	Not serious (sample >1,000, 95%CI does not cross 1)	Serious (asymmetrical funnel plot)	RR 0.49 (0.39-0.61)(+1)	Not reported	Moderate
Hofmeyr 2018	High dose calcium supplement on PE	13	SR	Serious risk of bias (high risk of attrition bias in L-Jaramillo 1989 and high risk of other bias in Kumar 2009)	Serious (1^2 76%)	Not serious (pregnant women)	Not serious (sample >1,000, 95%CI does not cross 1)	Serious (asymmetrical funnel plot)	RR 0.45 (0.31-0.65) (+1)	No; low dose calcium RR 0.38 (0.28-0.52)	Low
Hofmeyr 2018	Low dose calcium supplement on PE	9	SR	Very serious risk of bias (lack of blinding in Almirante 1998, Cong 1995, Marya 1987, Rogers 1999 and Taherian	Not serious (1^2 0%)	Not serious (pregnant women)	Not serious (sample >1,000, 95%CI does not cross 1)	NA (less than 10 studies included in meta-analysis)	RR 0.38 (0.28-0.52) (+1)	No (see for high dose calcium)	Moderate

				2002; high risk of selection bias in Rogers 1999 and Marya 1987; high risk of attrition bias Bassaw 1998, Rogers 1999; high risk of other bias in Almirante 1998, Cong 1995, Marya 1987, Rogers 1999 and Taherian 2002)							
Hofmeyr 2019	Peri-conception calcium supplement on PE	1	SR	Serious risk of bias (high risk of attrition bias in Hofmeyr 2019)	NA (only one included study)	Not serious (pre-conception or pregnant women)	Very serious (sample <1,000 and one trial only, 95%CI crosses 1)	NA (less than 10 studies included in meta-analysis)	RR 0.80 (0.61-1.06)	Not reported	Very low
Rumbold 2008	Any antioxidants supplement on PE	9	SR	No serious risk of bias (no studies rated with high risk of bias)	Serious (1^2 56%)	Not serious (pregnant women)	Serious (sample >1,000, 95%CI crosses 1)	NA (less than 10 studies included in meta-analysis)	RR 0.73 (0.51-1.06)	Not reported	Low
Salam 2015	Vitamin B6 (oral) supplement on PE	2	SR	Serious risk of bias (high risk of other bias in Swartwout 1960)	NA (only one included study with events)	Not serious (pregnant women)	Serious (sample >1,000, 95%CI crosses 1)	NA (less than 10 studies included in meta-analysis)	RR 1.71 (0.85-3.45)	Not reported	Low

Kinshella 2021	Vitamin C and/or E supplement on PE	19	UR	Not serious (majority studies low/unclear RoB)	Not serious (I^2 33%)	Not serious	Serious (large sample but 95%CI crosses 1)	Serious (asymmetrical funnel plot)	RR 0.96 (0.89-1.04	Not reported	Low
Rumbold 2015a	Vitamin C supplement on PE	16	SR	Serious risk of bias (lack of blinding in Kalpdev 2011 and Nasrolahi 2006; high risk of selection bias in Nasrolahi 2006)	Not serious (1^2 41%)	Not serious (pregnant women)	Serious (sample >1,000, 95%CI crosses 1)	Serious (asymmetrical funnel plot)	RR 0.92 (0.80-1.05)	Not reported	Very low
Rumbold 2015b	Vitamin E supplement on PE	14	SR	Serious risk of bias (lack of blinding in Kalpdev 2011 and Nasrolahi 2006; high risk of selection bias in Nasrolahi 2006; high risk of attrition bias in Huria 2010)	Not serious (1^2 48%)	Not serious (pregnant women)	Serious (sample >1,000, 95%CI crosses 1)	Serious (asymmetrical funnel plot)	RR 0.91 (0.79-1.06)	Not reported	Very low
Kinshella 2021	Iron and/or folic acid supplement on PE	6	UR	Serious (Less than half low/unclear RoB)	Not serious (I^2 0%)	Not serious	Serious (large sample but 95%CI crosses 1)	N/A	RR 0.99 (0.67-1.47)	Not reported	Low

Peña-Rosas 2015	Iron supplement on PE	4	SR	Serious risk of bias (high risk of attrition bias in Eskeland 1997)	Not serious (1^2 0%)	Not serious (pregnant women)	Serious (sample >1,000, 95%CI crosses 1)	NA (less than 10 studies included in meta-analysis)	RR 1.63 (0.87-3.07)	Not reported	Low
Peña-Rosas 2015	Iron and folic acid supplement on PE	1	SR	Serious risk of bias (lack of blinding of participants and personnel in Taylor 1982)	NA (only one included study)	Not serious (pregnant women)	Very serious (sample <1,000 and one trial included only, 95%CI crosses 1)	NA (less than 10 studies included in meta-analysis)	RR 3.0 (0.13-70.16)	Not reported	Very low
Yuan 2022	Magnesium supplement on PE	7	SR	Serious risk of bias (high risk of selection bias in Spatling 1988)	Not serious (1^2 0%)	Not serious (pregnant women)	Not serious (sample >1,000, 95%CI does not cross 1)	NA (less than 10 studies included in meta-analysis)	RR 0.76 (0.59-0.98)	Not reported	Moderate
Kinshella 2021, UR	Zinc supplement on PE	5	RCTs	Serious (Less than half low/unclear RoB)	Not serious (I^2 39%)	Not serious (pregnant women)	Serious (large sample but 95%CI crosses 1)	NA (less than 10 studies included in meta-analysis)	RR 1.31 (0.82-2.10)	Not reported	Low
Ota 2015b	Zinc supplement on PE/PIH	7	SR	Serious risk of bias (high risk of attrition bias in Xie 2001, Jonsson 1996; high risk of reporting bias in Hunt 1985)	Not serious (1^2 26%)	Not serious (pregnant women)	Serious (sample >1,000, 95%CI crosses 1)	NA (less than 10 studies included in meta-analysis)	RR 0.83 (0.64-1.08)	Not reported	Low
Kinshella 2021, UR	Multiple micronutrient supplemnetation on PE	2	RCTs	Serious (Less than half low/unclear RoB)	Not serious (I^2 0%)	Not serious (pregnant women)	Serious (sample <1,000, 95%CI does not cross 1)	NA (less than 10 studies included in meta-analysis)	RR 0.40 (0.27–0.59)	Not reported	Low

Kinshella 2021	Omega-3 supplement on PE	18	RCTs	Not serious (Majority studies low/unclear RoB)	Not serious (I^2 9%)	Not serious	Serious (large sample but 95%CI crosses 1)	Not serious (symmetrical funnel plot)	RR 0.87 (0.71-1.07)	Not reported	Moderate
Middleton 2019	Omega 3 supplement on PE	20	SR	Serious risk of bias (high risk of selection bias in Mardones 2008; high risk of attrition bias in Harris 2015, Horvaticek 2017, Mardones 2008, Onwude 1995 and smuts 2003b; high risk of reporting bias in Horvaticek 2017 and Rivas-Echeverria 2000)	Not serious (1^2 13%)	Not serious (pregnant women)	Serious (sample >1,000, 95%CI crosses 1)	Not serious (symmetrical funnel plot)	RR 0.84 (0.69-1.01)	Not reported	Low
Ota 2015a	Balanced protein-energy supplement on PE	2	SR	Serious risk of bias (lack of blinding of participants and personnel in Girja 1984 and Mora 1978)	NA (only one included study with events)	Not serious (pregnant women)	Serious (sample <1,000 but SR, 95%CI crosses 1)	NA (less than 10 studies included in meta-analysis)	RR 1.48 (0.82-2.66)	Not reported	Low

Kinshella 2021	Antenatal dietary counselling on PE	15	RCTs	Not serious (Majority studies low/unclear RoB)	Not serious (I^2 14%)	Not serious	Serious (large sample but 95%CI crosses 1)	Not serious (symmetrical funnel plot)	RR 0.97 (0.82-1.13)	Not reported	Moderate
Kibret 2018	Healthy maternal diet on PE	4	SR	No serious risk of bias (No studies rated low quality on Academy of Nutrition and Dietetics quality appraisal tool)	Not serious (1^2 39%)	Not serious (pregnant women)	Not serious (sample >1,000, 95%CI does not cross 1)	NA (less than 10 studies included in meta-analysis)	OR 0.78 (0.70-0.86)	Not reported	High
Paula 2022	Ultra-processed foods-rich diet on PE	4	SR	Not serious (No studies rated low quality using the Joanna Briggs Institute Critical Appraisal tools)	Not serious (1^2 0%)	Not serious (pregnant women)	Not serious (sample >1,000, 95%CI does not cross 1)	NA (less than 10 studies included in meta-analysis)	OR 1.28 (1.15 – 1.42)	Not reported	High
Minhas 2022	Mediterranean style diet on PE	1	Observa-tional study > 1000 participants (Cohort)	Not serious (adjusted for potential confounders and sensitivity analysis)	NA (not a meta-analysis)	Not serious (pregnant women)	Not serious (sample >1,000, 95%CI does not cross 1)	NA (not a meta-analysis)	OR tertile 3 vs tertile 1 0.78 (0.64 – 0.96)	No; tertile 2 0.72 (0.59-0.89)	Low
Peña-Rosas 2015	Iron supplement on maternal anaemia	14	SR	Serious risk of bias (lack of blinding in Holly 1955, Pritchard 1958, Puolakka 1980; high risk of	Serious (1^2 80%)	Not serious (pregnant women)	Not serious (sample >1,000, 95%CI does not cross 1)	Not serious (symmetrical funnel plot)	RR 0.30 (0.19-0.46) (+1)	Not reported	Moderate

				selection bias in Chanarin 1971; high risk of attrition bias in Batu 1976, Eskeland 1997; high risk of reporting bias in Batu 1976; high risk of other bias in Pritchard 1958)							
Peña-Rosas 2015	Iron folic acid supplement on maternal anaemia	3	SR	Serious risk of bias (high risk of attrition and reporting bias in Batu 1976)	Not serious (1^2 0%)	Not serious (pregnant women)	Not serious (sample >1,000, 95%CI does not cross 1)	N/A (less than 10 studies included in meta-analysis)	RR 0.34 (0.21-0.54) (+1)	Not reported	High
Lassi 2013	Folic acid supplement on maternal anaemia	8	SR	Serious risk of bias (high risk of selection bias in Iyengar 1975, Menon 1962, Rae 1970, Willoughy 1967 and high risk of attrition bias in Batu 1976)	Serious (1^2 90%)	Not serious (pregnant women)	Serious (sample >1,000, 95%CI crosses 1)	N/A (less than 10 studies included in meta-analysis)	RR 0.62 (0.35-1.10)	Not reported	Very low

Buppasiri 2015	Calcium supplement on maternal anaemia	1	SR	No serious risk of bias (no studies rated with high risk of bias)	N/A (only one included study)	Not serious (pregnant women)	Very serious (sample <1,000 and 1 trial with events included only, 95%CI crosses 1)	N/A (less than 10 studies included in meta-analysis)	RR 1.04 (0.90-1.22)	Not reported	Low
Middleton 2019	Omega 3 supplement on maternal anaemia	1	SR	No serious risk of bias (no studies rated with high risk of bias)	N/A (only one included study)	Not serious (pregnant women)	Very serious (sample <1,000 and 1 trial included only, 95%CI crosses 1)	N/A (less than 10 studies included in meta-analysis)	RR 1.16 (0.91-1.48)	Not reported	Low
McCauley 2015	Vitamin A supplement on maternal anaemia	3	SR	No serious risk of bias(no studies rated with high risk of bias)	Serious (1^2 68%)	Not serious (pregnant women)	Not serious (sample >1,000, 95%CI does not cross 1)	N/A (less than 10 studies included in meta-analysis)	RR 0.64 (0.43-0.94)	Not reported	Moderate
Tripathi 2019	Maternal vitamin D on GDM	36	SR	Serious risk of bias (sensitivity analyses completed but not quality assessment of included studies)	Serious (1^2 73%)	Not serious (pregnant women)	Not serious (sample >1,000, 95%CI does not cross 1)	Not serious (symmetrical funnel plot, Begg test showed no significant publication bias)	OR 1.43 (1.23–1.67)	Not reported	Low
Kouroglou 2019	Maternal vitamin B12 on GDM	2	SR	No serious risk of bias (no studies rated low quality on NOS)	Not serious (1^2 0%)	Not serious (pregnant women)	Not serious (sample >1,000, 95%CI does not cross 1)	N/A (less than 10 studies included in meta-analysis)	OR 1.81 (1.25–2.63)	Not reported	High

Palacios 2019	Vitamin D supplement on GDM	4	SR	Serious risk of bias (high risk of selection bias and attrition bias and lack of blinding of participants and personnel in Sablok 2015, high risk of other bias in Shahgheibi 2016)	Not serious (1^2 0%)	Not serious (pregnant women)	Not serious (sample <1,000 but SR, 95%CI does not cross 1)	N/A (less than 10 studies included in meta-analysis)	RR 0.51 (0.27-0.97)	Not reported	Moderate
Palacios 2019	Vitamin D and calcium on GDM	1	SR	Serious risk of bias (lack of blinding in Asemi 2012)	N/A (only one included study)	Not serious (pregnant women)	Very serious (sample <1,000 and 1 trial included only, 95%CI crosses 1)	N/A (less than 10 studies included in meta-analysis)	RR 0.33 (0.01-7.84)	Not reported	Very low
Middleton 2019	Omega 3 supplement on GDM	12	SR	Serious risk of bias (lack of blinding in Hauner 2012; high risk of attrition bias in Hauner 2012, Min 2014, Smuts 2003b and van Goor 2009; high risk of reporting bias in Haghiac 2015)	Not serious (1^2 0%)	Not serious (pregnant women)	Serious (sample >1,000, 95%CI crosses 1)	Not serious (symmetrical funnel plot)	RR 1.02 (0.83-1.26)	Not reported	Low

KIbret 2018	Healthy maternal dietary pattern on GDM	5	SR	No serious risk of bias (No studies rated low quality on Academy of Nutrition and Dietetics quality appraisal tool)	Serious (1^2 69%)	Not serious (pregnant women)	Not serious (sample >1,000, 95%CI does not cross 1)	NA (less than 10 studies included in meta-analysis)	OR 0.78 (0.56-0.99)	Not reported	Moderate
Paula 2022	Ulta-processed foods-rich diet on GDM	10	SR	Not serious (No studies rated low quality using the Joanna Briggs Institute Critical Appraisal tools)	Serious (1^2 83%)	Not serious (women not excluded)	Not serious (sample >1,000, 95%CI does not cross 1)	Serious (asymmetrical funnel plot	OR 1.48 (1.17 – 1.87)	Not reported	Low
Yao 2015	Vitamin D deficiency on obesity	15	SR	Serious risk of bias (sensitivity analyses but no assessment of individual study quality)	Serious (1^2 81%)	Not serious (women not excluded)	Not serious (sample >1,000, 95%CI does not cross 1)	Serious (asymmetrical funnel plot	OR 3.43 (2.33-5.06) (+1)	Not reported	Low
Scholing 2018	Pre-pregnancy obesity on folate deficiency	1	Observa-tional study > 1000 participants (Cohort)	Not serious (adjusted for potential confounders and sensitivity analysis)	NA (not a meta-analysis)	Not serious (pregnant women)	Not serious (sample >1,000, 95%CI does not cross 1)	NA (not a meta-analysis)	aOR 2.03 (1.35-3.06) (+1)	Yes: obesity had larger effect than overweight (+1)	High

Scholing 2018	Pre-pregnancy obesity on ferritin deficiency	1	Observa-tional study > 1000 participants (Cohort)	Not serious (adjusted for potential confounders and sensitivity analysis)	NA (not a meta-analysis)	Not serious (pregnant women)	Serious (sample >1,000 but 95%CI crosses 1)	NA (not a meta-analysis)	aOR 0.96 (0.62-1.51)	No	Very low
Scholing 2018	Pre-pregnancy obesity on serum iron deficiency	1	Observa-tional study > 1000 participants (Cohort)	Not serious (adjusted for potential confounders and sensitivity analysis)	NA (not a meta-analysis)	Not serious (pregnant women)	Not serious (sample >1,000, 95%CI does not cross 1)	NA (not a meta-analysis)	aOR 3.26 (2.09-5.08) (+1)	Yes: obesity had larger effect than overweig ht (+1)	High
Scholing 2018	Pre-pregnancy obesity on serum vitamin B12 deficiency	1	Observa-tional study > 1000 participants (Cohort)	Not serious (adjusted for potential confounders and sensitivity analysis)	NA (not a meta-analysis)	Not serious (pregnant women)	Not serious (sample >1,000, 95%CI does not cross 1)	NA (not a meta-analysis)	aOR 2.05 (1.41-2.99) (+1)	Yes: obesity had larger effect than overweig ht (+1)	High
Laraia 2007	Pre-pregnancy obesity on low diet quality during pregnancy	1	Observa-tional study > 1000 participants (Cohort)	Not serious (adjusted for potential confounders)	NA (not a meta-analysis)	Not serious (pregnant women)	Not serious (sample >1,000, 95%CI does not cross 1)	NA (not a meta-analysis)	aOR 1.76 (1.24–2.49)	Yes: dose response described (+1)	Moderate
Scholing 2018	Pre-pregnancy overweight on folate deficiency	1	Observa-tional study > 1000 participants (Cohort)	Not serious (adjusted for potential confounders and sensitivity analysis)	NA (not a meta-analysis)	Not serious (pregnant women)	Not serious (sample >1,000, 95%CI does not cross 1)	NA (not a meta-analysis)	aOR 1.38 (1.03-1.85)	Yes: obesity had larger effect than overweig ht (+1)	Moderate

Scholing 2018	Pre-pregnancy overweight on ferritin deficiency	1	Observa-tional study > 1000 participants (Cohort)	Not serious (adjusted for potential confounders and sensitivity analysis)	NA (not a meta-analysis)	Not serious (pregnant women)	Serious (sample >1,000 but 95%CI crosses 1)	NA (not a meta-analysis)	aOR 0.96 (0.73-1.27)	No	Very low
Scholing 2018	Pre-pregnancy overweight on serum iron deficiency	1	Observa-tional study > 1000 participants (Cohort)	Not serious (adjusted for potential confounders and sensitivity analysis)	NA (not a meta-analysis)	Not serious (pregnant women)	Not serious (sample >1,000, 95%CI does not cross 1)	NA (not a meta-analysis)	aOR 1.45 (1.02-2.07)	Yes: obesity had larger effect than overweig ht (+1)	Moderate
Scholing 2018	Pre-pregnancy overweight on serum vitamin B12 deficiency	1	Observa-tional study > 1000 participants (Cohort)	Not serious (adjusted for potential confounders and sensitivity analysis)	NA (not a meta-analysis)	Not serious (pregnant women)	Not serious (sample >1,000, 95%CI does not cross 1)	NA (not a meta-analysis)	aOR 1.38 (1.08-1.77)	Yes: obesity had larger effect than overweig ht (+1)	Moderate
Laraia 2007	Pre-pregnancy overweight on low diet quality during pregnancy	1	Observa-tional study > 1000 participants (Cohort)	Not serious (adjusted for potential confounders)	NA (not a meta-analysis)	Not serious (pregnant women)	Serious (sample >1,000 but 95%CI crosses 1)	NA (not a meta-analysis)	aOR 1.31 (0.87–1.99)	Yes: dose response described (+1)	Low
Tan 2019	Maternal vitamin D deficiency on maternal depression	6	SR	No serious risk of bias (no studies rated low quality on NOS)	Serious (1^2 82%)	Not serious (pregnant women)	Not serious (sample >1,000, 95%CI does not cross 1)	Not serious (symmetrical funnel plot, Begg and Eggers test showed no significant publication bias)	OR 0.49 (0.35-0.63) (+1)	Yes: the dose–response analysis indicated that the lowest pooled OR was at blood 25(OH)D concentr	High

										ations of 90–110 nmol/l (+1)	
Yee 2020	Healthy Eating Index on chronic hypertensio n	1	Observa-tional study > 1000 participants (Cohort)	Serious risk of bias (this association was evaluated as bivariate analyses and not adjusted for potential confounders)	NA (not a meta-analysis)	Not serious (pregnant women)	Not serious (sample >1,000, 95%CI does not cross 1)	NA (not a meta-analysis)	RR 2.67 (1.67-4.24) (+1)	Not reported	Low

PE – pre-eclampsia; PIH – pregnancy induced hypertension; GDM – gestational diabetes mellitus; SR – systematic review; NOS – Newcastle Ottawa Scale; OR – odds ratio; NA – not applicable

Table S3: Medical risk factors for pre-eclampsia incidence

Medical condition (definition)	Main reference author, year	Effect estimate (95% CI)	Number of studies	Number of participants	I^2	Strength of association*	Quality of evidence
Maternal anaemia (Hb <11g/dL, measured at any point during pregnancy) on pre-eclampsia	Young 2019	OR 1.84 (1.31–2.59) [1]	8	1,267,292	Not reported†	Probable	Low‡
Gestational diabetes mellitus (clinical diagnosis) on pre-eclampsia	Bilano 2014	aOR 2.00 (1.63–2.45)	1	276,388	N/A	Probable	Moderate§
Maternal overweight (BMI 25.0-29.9) on pre-eclampsia	Bartsch 2016	RR 2.1 (2.0-2.2) [1]	38	3,644,747	89%	Probable	High¶
Maternal obesity (BMI ≥30) on pre-eclampsia	Bartsch 2016	RR 2.8 (2.6-3.1) [1]	40	5,921,559	90%	Probable	High¶
Antenatal maternal depression (clinical diagnosis) on pre-eclampsia	Hu 2015	OR 1.63 (1.32-2.02) [1]	5	3,979	16%	Probable	High#
Chronic hypertension (pre-existing or hypertension diagnosed before 20 weeks) on pre-eclampsia	Bartsch 2016	RR 5.1 (4.0-6.5) [1]	20	6,589,661	98%	Definite	High**

[1] random effects model

[2] fixed effects model

* Definite (RR<0.40 or ≥3.00), probable (RR 0.40-0.69 or 1.50-2.99), possible (RR 0.70-0.89 or 1.10-1.49) or not discernible/not significant (RR 0.90-1.09) (15,20).

† Noted that most analyses showed significant heterogeneity but I^2 not reported

‡ Downgraded for lack of sensitivity analyses or quality assessment (potential risk of bias), I^2 not reported (potential risk of inconsistency) and no investigation of publication bias; upgraded for potential dose response

§ Starting point at low quality of evidence (single observational study), upgraded for large effect

¶ Downgraded for lack of assessment of individual study quality beyond attrition (potential risk of bias), high heterogeneity; upgraded for large effect size and evidence of dose-response

Starting point at high quality of evidence (systematic review), no downgrading

** Downgraded for lack of assessment of individual study quality beyond attrition (potential risk of bias), high heterogeneity; upgraded for very large effect size

Table S4: Nutritional factors with direct associations with pre-eclampsia development, by strength of association and certainty of evidence

Strength of association		Quality of evidence (GRADE)*		
		**High**	**Moderate**	**Low**
	**Definite**	• Maternal vitamin D deficiency (risk factor)<br>• Higher serum iron status (risk factor)<br>• Higher serum zinc status (protective)	• Higher serum vitamin C status (protective)	
	**Probable**		• Higher serum vitamin E status (protective)<br>• Vitamin D supplementation (protective)<br>• Vitamin D and calcium supplementation (protective)<br>• Calcium supplementation (protective)	• Multiple micronutrient supplementation (protective)
	**Possible**	• Healthy maternal dietary pattern (protective)<br>• Ultra-processed foods dietary pattern (risk factor)	• Magnesium supplementation (protective)	• Lower maternal serum vitamin B12 (risk factor)†<br>• Lower maternal serum selenium (risk factor)†

*No significantly associated nutritional factors were rated very low quality in our evidence review

† Lower levels among pre-eclamptic women in comparison to controls; odds/risk ratios not available
